# Mapping the smoking addiction using dynamic causal modelling at rest

**DOI:** 10.1186/1471-2202-16-S1-P246

**Published:** 2015-12-18

**Authors:** Rongxiang Tang, Adeel Razi, Yi-Yuan Tang

**Affiliations:** 1 Department of Psychology, The University of Texas at Austin, Austin, TX 78712, USA; 2 The Wellcome Trust Centre for Neuroimaging, University College London, London WC1N 3BG, UK; 3 Department of Psychological Sciences, Texas Tech University, Lubbock, TX 79409, USA

## 

Tobacco use is the leading preventable cause of death. Previous research show that brain areas including medial prefrontal cortex (mPFC), posterior cingulate cortex (PCC) and others involved in smoking addiction [1]. However it remains unclear which brain regions play a crucial role in smoking addiction and the relationship among these regions. Since functional connectivity does not support inferences about causal brain connections, the changes in information flow in these distributed systems involved in smoking remain largely unknown. Here we apply a dynamic causal modeling (DCM) in resting state fMRI [2] to demonstrate the causal relationships among the core regions in smoking addiction.

Healthy college students were recruited through campus advertisements. Among those who responded, there were 14 cigarette smokers and 14 nonsmokers. All data were collected using a 3-Telsa Siemens Skyra scanner. Functional data were processed using the Data Processing Assistant for Resting-State fMRI, which is based on SPM (www.fil.ion.ucl.ac.uk/spm) and Resting-State fMRI Data Analysis Toolkit. Based on literature, we specified four regions of interest (ROIs) within the default mode network (DMN), which are medial prefrontal cortex (mPFC), posterior cingulate cortex (PCC), and bilateral inferior parietal lobule ( Left IPL and Right IPL), same regions and coordinates as in previous studies that use sDCM analysis for resting state [2,3]. Based on SPM12, we estimated and specified the DCM for each subject and later compared the differences of effective connectivity between two groups by using two-sample test. Our results suggest the different causal relationships between nonsmokers and smokers. Specifically, there was a lower strength of excitatory input from PCC to mPFC for smokers than nonsmokers and a lower strength of excitatory input from RIPL to mPFC for smokers than nonsmokers (all p<0.05).

## Conclusions

These data indicate the usage of DCM on resting-state fMRI data can differentiate the causal brain connections between two groups, and provide insight into the brain mechanisms underlying smoking addiction - the abnormalities of causal connectivity associated with attention and self-control networks in the brain. Our results may also suggest the brain based prevention and intervention should consider the amelioration of the mPFC-PCC circuits.
